# Supplementary material for: Evaluation of Listeria monocytogenes Dissemination in a Beef Steak Tartare Production Chain
Source: Foods. 2025 Sep 29;14(19):3372. doi: 10.3390/foods14193372 (PMC12524288; doi:10.3390/foods14193372)
Supplement: Supplementary file 1 [file foods-14-03372-s001.zip › foods-3886913-supplementary.pdf]

Table S1. Strain identification of 114 LM isolates and related information on sample, sampling session and site, clonal complex, sequence type, cgMLST cluster number, and Biosample accession number for each isolate.

Strain identification	Sample	Sampling session	Type of sample	Sampling site	Clonal complex (CC)	Sequence type (ST)	cgMLST cluster	NCBI Biosample
*Lm*1	St1/8	St1/1	litter	1° farm	CC70	ST70	Cluster I	SAMN48062199
*Lm*2	St1/8	St1/1	litter	1° farm	CC70	ST70	Cluster I	SAMN48062200
*Lm*3	St1/8	St1/1	litter	1° farm	CC70	ST70	Cluster I	SAMN48062201
*Lm*4	St1/8	St1/1	litter	1° farm	CC70	ST70	Cluster I	SAMN48062202
*Lm*5	St1/8	St1/1	litter	1° farm	CC70	ST70	Cluster I	SAMN48062203
*Lm*6	St1/9	St1/1	litter	1° farm	CC70	ST70	Cluster I	SAMN48062204
*Lm*7	St1/9	St1/1	litter	1° farm	CC70	ST70	Cluster I	SAMN48062205
*Lm*8	St1/9	St1/1	litter	1° farm	CC70	ST70	Cluster I	SAMN48062206
*Lm*9	St1/9	St1/1	litter	1° farm	CC70	ST70	Cluster I	SAMN48062207
*Lm*10	St1/9	St1/1	litter	1° farm	CC70	ST70	Cluster I	SAMN48062208
*Lm*11	St1/21	St1/1	animal skin	slaughterhouse	CC9	ST9	Cluster IV	SAMN48062209
*Lm*12	St1/21	St1/1	animal skin	slaughterhouse	CC9	ST9	Cluster IV	SAMN48062210
*Lm*13	St1/21	St1/1	animal skin	slaughterhouse	CC9	ST9	Cluster IV	SAMN48062211
*Lm*14	St1/21	St1/1	animal skin	slaughterhouse	CC9	ST9	Cluster IV	SAMN48062212
*Lm*15	St1/21	St1/1	animal skin	slaughterhouse	CC9	ST9	Cluster IV	SAMN48062213
*Lm*16	St1/26	St1/1	carcass	slaughterhouse	CC9	ST580	Cluster III	SAMN48062214
*Lm*17	St1/26	St1/1	carcass	slaughterhouse	CC9	ST580	Cluster III	SAMN48062215
*Lm*18	St1/26	St1/1	carcass	slaughterhouse	CC9	ST580	Cluster III	SAMN48062216
*Lm*19	St1/26	St1/1	carcass	slaughterhouse	CC9	ST580	Cluster III	SAMN48062217
*Lm*20	St1/26	St1/1	carcass	slaughterhouse	CC9	ST580	Cluster III	SAMN48062218
*Lm*21	St1/28	St1/1	carcass	slaughterhouse	CC9	ST580	Cluster III	SAMN48062219
*Lm*22	St1/28	St1/1	carcass	slaughterhouse	CC9	ST580	Cluster III	SAMN48062220
*Lm*23	St1/28	St1/1	carcass	slaughterhouse	CC9	ST580	Cluster III	SAMN48062221
*Lm*24	St1/28	St1/1	carcass	slaughterhouse	CC9	ST580	Cluster III	SAMN48062222
*Lm*25	St1/28	St1/1	carcass	slaughterhouse	CC9	ST580	Cluster III	SAMN48062223
*Lm*26	St1/29	St1/1	carcass	slaughterhouse	CC9	ST580	Cluster III	SAMN48062224
*Lm*27	St1/29	St1/1	carcass	slaughterhouse	CC9	ST580	Cluster III	SAMN48062225
*Lm*28	St1/29	St1/1	carcass	slaughterhouse	CC9	ST580	Cluster III	SAMN48062226

*Lm*29	St1/29	St1/1	carcass	slaughterhouse	CC9	ST580	Cluster III	SAMN48062227
*Lm*30	St1/29	St1/1	carcass	slaughterhouse	CC9	ST580	Cluster III	SAMN48062228
*Lm*31	St1/31	St1/1	carcass	slaughterhouse	CC9	ST580	Cluster III	SAMN48062229
*Lm*32	St1/31	St1/1	carcass	slaughterhouse	CC9	ST580	Cluster III	SAMN48062230
*Lm*33	St1/31	St1/1	carcass	slaughterhouse	CC9	ST580	Cluster III	SAMN48062231
*Lm*34	St1/31	St1/1	carcass	slaughterhouse	CC9	ST580	Cluster III	SAMN48062232
*Lm*35	St1/31	St1/1	carcass	slaughterhouse	CC9	ST580	Cluster III	SAMN48062233
*Lm*36	St1/36	St1/1	drains	slaughterhouse	CC9	ST580	Cluster III	SAMN48062234
*Lm*37	St1/36	St1/1	drains	slaughterhouse	CC9	ST580	Cluster II	SAMN48062235
*Lm*38	St1/41	St1/1	dicing machine	hamburger production	CC9	ST580	Cluster III	SAMN48062236
*Lm*39	St1/41	St1/1	dicing machine	hamburger production	CC9	ST580	Cluster III	SAMN48062237
*Lm*40	St1/41	St1/1	dicing machine	hamburger production	CC9	ST580	Cluster III	SAMN48062238
*Lm*41	St1/41	St1/1	dicing machine	hamburger production	CC9	ST580	Cluster III	SAMN48062239
*Lm*42	St1/41	St1/1	dicing machine	hamburger production	CC9	ST580	Cluster III	SAMN48062240
*Lm*43	St1/42	St1/1	drains	hamburger production	CC9	ST580	Cluster III	SAMN48062241
*Lm*44	St1/42	St1/1	drains	hamburger production	CC9	ST580	Cluster III	SAMN48062242
*Lm*45	St1/72	St1/2	drains	hamburger production	CC9	ST580	Cluster III	SAMN48062243
*Lm*46	St1/72	St1/2	drains	hamburger production	CC9	ST580	Cluster III	SAMN48062244
*Lm*47	St1/72	St1/2	drains	hamburger production	CC9	ST580	Cluster III	SAMN48062245
*Lm*48	St1/72	St1/2	drains	hamburger production	CC9	ST580	Cluster III	SAMN48062246
*Lm*49	St1/73	St1/2	animal skin	slaughterhouse	CC9	ST580	Cluster III	SAMN48062247
*Lm*50	St1/81	St1/2	carcass	slaughterhouse	CC9	ST580	Cluster II	SAMN48062248
*Lm*51	St1/81	St1/2	carcass	slaughterhouse	CC9	ST580	Cluster II	SAMN48062249
*Lm*52	St1/84	St1/2	carcass	slaughterhouse	CC9	ST580	Cluster II	SAMN48062250
*Lm*53	St1/86	St1/2	carcass	slaughterhouse	CC9	ST580	Cluster II	SAMN48062251
*Lm*54	St1/86	St1/2	carcass	slaughterhouse	CC9	ST580	/	SAMN48062252
*Lm*55	St1/86	St1/2	carcass	slaughterhouse	CC9	ST580	Cluster II	SAMN48062253
*Lm*56	St1/86	St1/2	carcass	slaughterhouse	CC9	ST580	Cluster II	SAMN48062254
*Lm*57	St1/86	St1/2	carcass	slaughterhouse	CC9	ST3286	/	SAMN48062255
*Lm*58	St1/86	St1/2	carcass	slaughterhouse	CC9	ST580	Cluster II	SAMN48062256

*Lm*59	St1/87	St1/2	drains	slaughterhouse	CC9	ST580	Cluster II	SAMN48062257
*Lm*60	St1/87	St1/2	drains	slaughterhouse	CC9	ST580	Cluster II	SAMN48062258
*Lm*61	St1/88	St1/2	drains	slaughterhouse	CC9	ST580	Cluster III	SAMN48062259
*Lm*62	St1/90	St1/2	drains	slaughterhouse	CC6	ST6	/	SAMN48113846
*Lm*63	St1/92	St1/2	drains	hamburger production	CC121	ST121	/	SAMN48062260
*Lm*64	St3/14	St3	meat-cubed	steak tartare production	CC9	ST580	Cluster III	SAMN48530348
*Lm*65	St3/15	St3	meat-cubed	steak tartare production	CC9	ST580	Cluster II	SAMN48530349
*Lm*66	St3/15	St3	meat-cubed	steak tartare production	CC2	ST145	/	SAMN48530350
*Lm*67	St3/18	St3	meat-cubed	steak tartare production	CC9	ST580	Cluster III	SAMN48530351
*Lm*68	St3/18	St3	meat-cubed	steak tartare production	CC9	ST580	Cluster III	SAMN48530352
*Lm*69	St3/18	St3	meat-cubed	steak tartare production	CC9	ST580	Cluster III	SAMN48530353
*Lm*70	St3/18	St3	meat-cubed	steak tartare production	CC9	ST580	Cluster III	SAMN48530354
*Lm*71	St3/19	St3	meat-cubed	steak tartare production	CC9	ST580	Cluster III	SAMN48530355
*Lm*72	St3/19	St3	meat-cubed	steak tartare production	CC9	ST580	Cluster III	SAMN48530356
*Lm*73	St3/19	St3	meat-cubed	steak tartare production	CC9	ST580	Cluster III	SAMN48530357
*Lm*74	St3/2	St3	primal cut	sectioning	CC9	ST580	Cluster II	SAMN48530358
*Lm*75	St3/21	St3	meat-formed	steak tartare production	CC9	ST580	Cluster III	SAMN48530359
*Lm*76	St3/21	St3	meat-formed	steak tartare production	CC9	ST580	Cluster III	SAMN48530360
*Lm*77	St3/21	St3	meat-formed	steak tartare production	CC9	ST580	Cluster III	SAMN48530361
*Lm*78	St3/22	St3	meat-hardened	steak tartare production	CC9	ST580	Cluster III	SAMN48530362
*Lm*79	St3/22	St3	meat-hardened	steak tartare production	CC9	ST580	Cluster III	SAMN48530363
*Lm*80	St3/23	St3	meat-hardened	steak tartare production	CC9	ST580	Cluster III	SAMN48530364
*Lm*81	St3/24	St3	meat-hardened	steak tartare production	CC9	ST580	Cluster III	SAMN48530365
*Lm*82	St3/25	St3	meat-hardened	steak tartare production	CC9	ST580	Cluster III	SAMN48530366
*Lm*83	St3/26	St3	steak tartare final product	steak tartare production	CC9	ST580	Cluster III	SAMN48530367
*Lm*84	St3/27	St3	steak tartare final product	steak tartare production	CC9	ST580	Cluster III	SAMN48530368
*Lm*85	St3/28	St3	steak tartare final product	steak tartare production	CC9	ST580	Cluster III	SAMN48530369
*Lm*86	St3/29	St3	steak tartare final product	steak tartare production	CC9	ST580	Cluster III	SAMN48530370

*Lm*87	St3/4	St3	meat-peeled	steak tartare production	CC9	ST580	Cluster II	SAMN48530371
*Lm*88	St3/4	St3	meat-peeled	steak tartare production	CC9	ST580	Cluster III	SAMN48530372
*Lm*89	St3/5	St3	meat-peeled	steak tartare production	CC9	ST580	Cluster III	SAMN48530373
*Lm*90	St3/5	St3	meat-peeled	steak tartare production	CC9	ST580	Cluster III	SAMN48530374
*Lm*91	St3/5	St3	meat-peeled	steak tartare production	CC9	ST580	Cluster II	SAMN48530375
*Lm*92	St3/7	St3	meat-dressed	steak tartare production	CC9	ST580	Cluster III	SAMN48530376
*Lm*93	St3/7	St3	meat-dressed	steak tartare production	CC9	ST580	Cluster III	SAMN48530377
*Lm*94	St3/7	St3	meat-dressed	steak tartare production	CC9	ST580	Cluster II	SAMN48530378
*Lm*95	St3/7	St3	meat-dressed	steak tartare production	CC9	ST580	Cluster II	SAMN48530379
*Lm*96	St3/8	St3	meat-dressed	steak tartare production	CC9	ST580	Cluster II	SAMN48530380
*Lm*97	St3/10	St3	rack	steak tartare production	CC9	ST580	Cluster III	SAMN48530381
*Lm*98	St3/16	St3	mixing machine	steak tartare production	CC9	ST580	Cluster III	SAMN48530382
*Lm*99	St3/16	St3	mixing machine	steak tartare production	CC9	ST580	Cluster III	SAMN48530383
*Lm*100	St3/16	St3	mixing machine	steak tartare production	CC9	ST580	Cluster III	SAMN48530384
*Lm*101	St3/16	St3	mixing machine	steak tartare production	CC9	ST580	Cluster III	SAMN48530385
*Lm*102	St3/17	St3	tub	steak tartare production	CC9	ST580	Cluster III	SAMN48530386
*Lm*103	St3/20	St3	forming machine	steak tartare production	CC9	ST580	Cluster III	SAMN48530387
*Lm*104	St3/20	St3	forming machine	steak tartare production	CC9	ST580	Cluster III	SAMN48530388
*Lm*105	St3/20	St3	forming machine	steak tartare production	CC9	ST580	Cluster III	SAMN48530389
*Lm*106	St3/3	St3	peeling machine	steak tartare production	CC9	ST580	Cluster III	SAMN48530390
*Lm*107	St3/3	St3	peeling machine	steak tartare production	CC9	ST580	/	SAMN48530391
*Lm*108	St3/3	St3	peeling machine	steak tartare production	CC9	ST580	Cluster II	SAMN48530392
*Lm*109	St3/6	St3	dressing table	steak tartare production	CC9	ST580	Cluster II	SAMN48530393
*Lm*110	St3/20	St3	forming machine	steak tartare production	CC9	ST580	Cluster III	SAMN48530394
*Lm*111	St3/21	St3	meat-formed	steak tartare production	CC9	ST580	Cluster III	SAMN48530395
*Lm*112	St3/22	St3	meat-hardened	steak tartare production	CC9	ST580	Cluster III	SAMN48530396
*Lm*113	St3/29	St3	steak tartare final product	steak tartare production	CC9	ST580	Cluster III	SAMN48530397
*Lm*114	St3/1	St3	primal cut	sectioning	CC9	ST580	Cluster III	SAMN48530398

/ = no cluster observed.
